# Use of Maximum Likelihood-Mixed Models to select stable reference genes: a case of heat stress response in sheep

**DOI:** 10.1186/1471-2199-12-36

**Published:** 2011-08-17

**Authors:** Magdalena Serrano, Natalia Moreno-Sánchez, Carmen González, Ane Marcos-Carcavilla, Mario Van Poucke, Jorge H Calvo, Judit Salces, Jaime Cubero, María J Carabaño

**Affiliations:** 1 Departamento de Mejora Genética Animal, INIA, Ctra. de la Coruña km 7.5, Madrid, Spain; 2 Department of Nutrition, Genetics and Ethology, Faculty of Veterinary Medicine, Ghent University, Heidestraat 19, B-9820 Merelbeke, Belgium; 3 Unidad de Tecnología en Producción Animal, CITA, Zaragoza, Spain; 4 Departamento de Protección Vegetal, INIA. Ctra. de la Coruña km 7.5, Madrid, Spain

## Abstract

**Background:**

Reference genes with stable expression are required to normalize expression differences of target genes in qPCR experiments. Several procedures and companion software have been proposed to find the most stable genes. Model based procedures are attractive because they provide a solid statistical framework. NormFinder, a widely used software, uses a model based method. The pairwise comparison procedure implemented in GeNorm is a simpler procedure but one of the most extensively used. In the present work a statistical approach based in Maximum Likelihood estimation under mixed models was tested and compared with NormFinder and geNorm softwares. Sixteen candidate genes were tested in whole blood samples from control and heat stressed sheep.

**Results:**

A model including gene and treatment as fixed effects, sample (animal), gene by treatment, gene by sample and treatment by sample interactions as random effects with heteroskedastic residual variance in gene by treatment levels was selected using goodness of fit and predictive ability criteria among a variety of models. Mean Square Error obtained under the selected model was used as indicator of gene expression stability. Genes top and bottom ranked by the three approaches were similar; however, notable differences for the best pair of genes selected for each method and the remaining genes of the rankings were shown. Differences among the expression values of normalized targets for each statistical approach were also found.

**Conclusions:**

Optimal statistical properties of Maximum Likelihood estimation joined to mixed model flexibility allow for more accurate estimation of expression stability of genes under many different situations. Accurate selection of reference genes has a direct impact over the normalized expression values of a given target gene. This may be critical when the aim of the study is to compare expression rate differences among samples under different environmental conditions, tissues, cell types or genotypes. To select reference genes not only statistical but also functional and biological criteria should be considered. Under the method here proposed *SDHA/MDH1 *have arisen as the best set of reference genes to be used in qPCR assays to study heat shock in ovine blood samples.

## Background

Quantitative real-time PCR (qPCR) has become a widely used method for both quantitative and qualitative determination of molecular targets. Reliable quantitative expression measurements depend on controlling several parameters (initial sample amount, efficiency of cDNA synthesis, etc). Usually, these parameters are normalized by means of one or more reference gene(s) (RGs), whose expression is supposed to remain stable in all the tissues and cells or along the different conditions under investigation. However, since the expression of some if not all RGs varies depending on biological samples [1] the use of RGs is controversial. Lee and co-workers [2] proposed that all genes are differentially expressed in at least one biological context, so the expression of every gene would be context dependent. Therefore, following the indications proposed by the MIQE (Minimum Information for Publication of Quantitative Real-Time PCR Experiments) guidelines [3] the utility of chosen reference genes must be confirmed by each research group for every experimental setup [4].

The use of proper mathematical methods to estimate the expression stability of genes under specific conditions is one of the most critical points in the search of RGs. In the last years, some efforts have been made to determine the best way to estimate expression stability of candidate reference genes. Concerning the former, the Pair-Wise comparison method employed by geNorm [4], the most used software in the establishment of RGs, considers that all the samples belong to one group (e.g. tissue, treatment, environmental conditions, etc), and conversely the estimate of the expression stability ignores differences in gene expression level and gene expression variability across groups. On the other hand, Andersen et al. [5] proposed a model-based approach to identify RGs, by means of the NormFinder Visual Basic application for Microsoft Excel. This method estimates the intra- and inter-group variances for each gene and calculates a stability value by combining both sources of variation. Another model-based procedure is the method proposed by Szabo et al. [6] which also uses the intra- and inter-gene variation across groups but in a more solid statistical framework. In this case, the stability criterion is the Mean Square Error (MSE), a measure of variability around an intended value, the overall expression level of a gene. Abruzzo et al [7] compared several models including the fixed effect models used in [6] and [5] and models with random effects, showing preference for a model with random gene by group interaction. A model based procedure using mixed models and optimal statistical methods, such as Maximum Likelihood, to estimate inter- and intra-group variances would then be desirable.

Our biological example is the case of the heat-stress response in the ovine species. Variations in the environmental temperature usually stress the organism and may result in the evolution of adaptative genetic mechanisms to cope with extreme temperatures. At cellular level, heat-shock lead to changes in gene expression in most (if not all) cells as well in a variety of organs and tissues associated with the acclimation response. There are some studies about gene expression under heat stress situations in species such as Dinoflagellates [8], Shrimps [9] and Honey bees [10] in which *Cal, Rp-S4*, *SAM *and *Tub*; *RPS18; *and *rp49 *have been used as RGs, respectively. De Boer et al. [11] developed a study to detect RGs for various stress conditions in soil arthropods. They found *SDHA, YWHAZ *and *ACTB *among the most stable genes under heat stress conditions. However, the suitability of genes for qPCR normalization in the field of adaptation to different thermal conditions in mammals, and particularly in the ovine species, remains unsolved.

In this study, we propose a Maximum Likelihood (ML) Mixed model based approach to estimate the expression stability of 16 candidate RGs taking the heat stress response in the ovine species as example. A comparison with other classical approaches widely used in qPCR experiments was also performed. To test the impact of using alternative methods, normalization factors were calculated with the Delta Ct method for the reference genes selected with each approach and used to normalize some target genes.

## Results

### Gene expression and qPCR efficiencies

**Figure 1:**
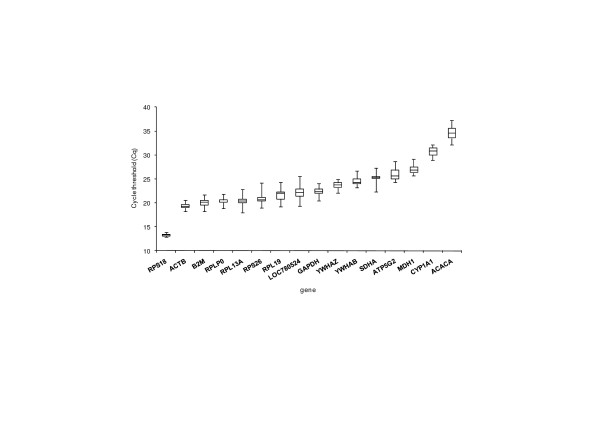
**Distribution of Cycle threshold (Cq) values for the candidate reference genes**. Distribution of Cycle threshold (Cq) values for the candidate reference genes (n = 29 samples) obtained by qPCR. Data of the three replicates per sample were averaged and those of the two thermal treatments were pooled for each gene. Boxes show the range of Cq values within each gene; the centre line indicates de median; extended vertical bars show standard deviation of the mean.

Figure 1 shows the distribution of the Quantification Cycle (Cq) values for the 16 genes tested. The highest expression rate was found for *RPS18*, with an average Cq value of 13.3. On the contrary, *ACACA *had the highest average Cq value (34.6), which indicated its low expression rate in leukocytes.

qPCR efficiencies (E) of the candidate RGs are shown in Table 1. Due to technical difficulties, *CYP1A1 *efficiency could not be estimated and its value (1.99) was taken from the literature [12]. Amplification E ranged from 74% to 98%. Higher E were found for candidate genes included in the Sheep geNorm kit (from 1.84 to 1.98), since primers and the PCR conditions must be designed to amplify sequences with E close to 2. Genes from Lampo et al. [13] showed lower E (from 1.74 to 1.77) probably because their PCR amplification conditions are different from the standard ones of the kit. Most qPCR studies use the log_2 _transformed Cycle Threshold or Crossing Point (named Cq according to MIQE guidelines [3]), as the expression rate variable, assuming that the E is maximum (100%) and therefore E = 2 for all genes. However, Tichopad et al. [14] pointed out that E evaluation is an essential marker in real-time quantification procedure and therefore corrected models by this parameter are highly recommended.

**Table 1:** Estimated gene amplification efficiencies

Gene	slope	Efficiency	correlation
*RPS18*	-3.593	1.90	1.000

*YWHAZ*	-3.377	1.98	0.995

*ACACA*	-3.701	1.86	0.999

*RPS26*	-3.701	1.86	0.999

*B2M*	-3.661	1.88	0.999

*MDH1*	-3.730	1.85	0.856

*LOC780524*	-3.598	1.90	0.998

*RPL19*	-3.648	1.88	0.842

*ATP5G2*	-3.775	1.84	0.993

*GAPDH*	-3.624	1.89	0.995

*CYP1A1*	-	1.99	0.997

*ACTB*	-3.727	1.85	0.998

*RPL13A*	-4.020	1.77	0.996

*RPLP0*	-4.161	1.74	0.997

*SDHA*	-4.033	1.77	0.996

*YWHAB*	-4.138	1.74	0.996

### Maximum Likelihood-Mixed model approach

Goodness of fit criteria (-2logL (L likelihood), AIC (Akaike's Information Criterion) and BIC (Schwarz's Bayesian Criterion)) and predictive ability (PD) values of the alternative mixed models tested are shown in Table 2. Full models including the three main effects (treatment, gene and sample) and all first order interactions(models 1, 2 and 3 in Table 3) showed both, better goodness of fit and lower PD than models that do not considers any of the interactions. This indicates that such interactions were relevant in fitting and predicting expression data obtained from different samples and treatments. Model 3 which considered heteroskedastic residual variance linked to the txg effect (32 levels), showed the best goodness of fit (BIC relative to minimum value = 100%; larger is better) and higher PD (PD relative to maximum = 60.1%; smaller is better) than models 1 and 2. Still model 3 showed lower PD than the reduced models (models 4 to 7). Model 2, which only differed from model 3 in the residual variance definition (in this case heteroskedastic residual variance was linked to the gene effect, 16 levels), yielded also good fit and predictive parameters (BIC% = 95.6; PD% = 52.5). On the other hand, model 1, with homoskedatic residual variance, had the worst goodness of fit (BIC% = 87.7) and was similar to model 2 in PD (PD% = 52.7). Model 8, which mimics the approach proposed by Andersen et al. [5] showed good behavior in fitting data (BIC% = 90.04) but very large predicting ability (PD% = 89.02).

**Table 2:** Goodness of fit and predictive ability criterion of the tested ML-mixed models

MID	n° parameters	-2LogL	AIC	BIC	BIC(%)	PD	PD(%)
1	5	-3141.0	-3097.0	-3081.4	87.67	0.001233	52.67

2	20	-3460.4	-3386.4	-3360.2	95.60	0.001230	52.54

3	36	-3658.2	-3552.2	-3514.7	100	0.001408	60.14

4	19	-3156.9	-3084.9	-3059.5	87.04	0.002027	86.58

5	35	-3203.0	-3099.0	-3062.2	87.12	0.002020	86.28

6	19	-3016.5	-2944.5	-2919.1	83.05	0.001827	78.04

7	35	-3308.0	-3204.0	-3167.2	90.11	0.002341	100

8	32	-3476.8	-3296.8	-3164.9	90.04	0.002084	89.02

MID = model identification, -2LogL = -2 log of the likelihood function (smaller is better), AIC = Akaike Information Criterion (smaller is better), BIC = Bayes Information Criterion (smaller is better), BIC(%) = percentage of fit (higher is better), PD = predictive ability criterion (smaller is better), PD(%) = percentage of predictive ability loss (smaller is better)

**Table 3:** ML-Mixed models description

MID	Model	Variance structure	n° (co)variance parameters
1	y_ijk _= μ + t_i _+ g_j _+ a_k _+ tg_ij _+ ta_ik _+ ga_jk _+ e_ijk_	a~N(0,σ^2^_a_); tg~N(0,σ^2^_tg_); ta~N(0,σ^2^_ta_); ga~N(0,σ^2^_ga_); e~N(0,σ^2^e)	5

2	y_ijk _= μ + t_i _+ g_j _+ a_k _+ tg_ij _+ ta_ik _+ ga_jk _+ e_ijk_	a~N(0,σ^2^_a_); tg~N(0,σ^2^_tg_); ta~N(0,σ^2^_ta_); ga~N(0,σ^2^_ga_); e~N(0,σ^2^e_j_)	20

3	y_ijk _= μ + t_i _+ g_j _+ a_k _+ tg_ij _+ ta_ik _+ ga_jk _+ e_ijk_	a~N(0,σ^2^_a_); tg~N(0,σ^2^_tg_); ta~N(0,σ^2^_ta_); ga~N(0,σ^2^_ga_); e~N(0,σ^2^e_ij_)	36

4	y_ijk _= μ + t_i _+ g_j _+ a_k _+ tg_ij _+ ta_ik _+ e_ijk_	a~N(0,σ^2^_a_); tg~N(0,σ^2^_tg_); ta~N(0,σ^2^_ta_); e~N(0,σ^2^e_j_)	19

5	y_ijk _= μ + t_i _+ g_j _+ a_k _+ tg_ij _+ ta_ik _+ e_ijk_	a~N(0,σ^2^_a_); tg~N(0,σ^2^_tg_); ta~N(0,σ^2^_ta_); e~N(0,σ^2^e_ij_)	35

6	y_ijk _= μ + t_i _+ g_j _+ a_k _+ tg_ij _+ ga_ik _+ e_ijk_	a~N(0,σ^2^_a_); tg~N(0,σ^2^_tg_); ga~N(0,σ^2^_ga_); e~N(0,σ^2^e_j_)	19

7	y_ijk _= μ + t_i _+ g_j _+ a_k _+ tg_ij _+ ga_ik _+ e_ijk_	a~N(0,σ^2^_a_); tg~N(0,σ^2^_tg_); ga~N(0,σ^2^_ga_); e~N(0,σ^2^e_ij_)	35

8	y_ijk _= μ + tg_ij _+ ta_ik _+ e_ijk_	e~N(0,σ^2^e_ij_)	32

In the Model column the fixed effects were t = treatment (2 levels) and g = gene (16 levels); the random effects were a = sample (15 levels), tg = interaction treatment × gene (32 levels), ta = interaction treatment × sample (29 levels), ga = interaction gene × sample (240 levels), and e = residual. Residual variances: σ^2^e = homoskedastic residuals and σ^2^e_j _= heteroskedastic residual for the gene effect (16 residual variances); σ^2^e_ij _= heteroskedastic residual for the treatment × gene effect (32 residual variances). Model 8 was proposed by Andersen et al [5], where txg y txa interactions are treated as fixed effects.

MID - model identification

Considering these results, models 2 and 3 were selected to estimate the stability value (MSE) of the 16 candidate RGs. For each gene, two MSE values, one for each treatment, were calculated. Table 4 shows estimates of bias, variances and MSE of the candidate RGs obtained with mixed model 3. Since results from models 2 and 3 were similar, only those from model 3 are shown. *RPS18 *and *B2M *showed the lowest bias, (small deviation of the expression in each treatment from the overall expression of the gene) which is indicative of the stability of these genes under heat stress conditions. On the contrary, *LOC780524, RPL19 *and *ATP5G2*, showed the highest bias values. Although some genes (*ATP5G2, GAPDH *and *ACTB*) showed higher variances in the control than in the stressed samples, or in the other way around (*RPL13A *and *RPLP0*), the overall variances of gene expression under both control and heat-stress were small, as expected for candidate RGs.

**Table 4:** Estimates of bias, variances and MSE of candidate reference genes obtained by fitting ML-mixed model 3

Gene		Variance	Variance	MSE	MSE	MSE
	
	Bias	Control	Heat stress	Control	Heat stress	max
*RPS18*	0.00652	0.00063	0.00002	0.00067	0.00006	0.00067

*RPS26*	0.01708	0.00059	0.00006	0.00088	0.00035	0.00088

*SDHA*	-0.01632	0.00006	0.00087	0.00033	0.00114	0.00114

*B2M*	0.00753	0.00008	0.00143	0.00014	0.00148	0.00148

*ACTB*	-0.02230	0.00118	0.00002	0.00168	0.00052	0.00168

*MDH1*	0.03549	0.00065	0.00009	0.00191	0.00135	0.00191

*YWHAZ*	-0.01932	0.00158	0.00069	0.00196	0.00106	0.00196

*LOC780524*	0.04607	0.00060	0.00007	0.00272	0.00219	0.00272

*GAPDH*	-0.02412	0.00243	0.00002	0.00302	0.00060	0.00302

*YWHAB*	-0.04385	0.00010	0.00139	0.00203	0.00331	0.00331

*RPLP0*	-0.01210	0.00009	0.00362	0.00023	0.00377	0.00377

*RPL19*	0.05618	0.00002	0.00073	0.00318	0.00389	0.00389

*ATP5G2*	0.06370	0.00163	0.00004	0.00569	0.00410	0.00569

*CYP1A1*	-0.02738	0.00188	0.00553	0.00263	0.00628	0.00628

*ACACA*	-0.02782	0.00613	0.00409	0.00691	0.00486	0.00691

*RPL13A*	-0.03935	0.00005	0.00628	0.00160	0.00783	0.00783

Bias of the control samples. Estimates of bias of the heat-stress samples were the same as for control but with opposite sign.

To rank genes on the basis of their stability, the minimax MSE criterion of Szabo et al. [6] was applied. Briefly, the gene with the minimum MSE value was selected as the most stable. The MSE value for each gene was the largest of the two MSE values for each treatment. Overall, *RPS18 *was the most stable gene and *RPL13A *the least.

### Comparison with geNorm and NormFinder

Table 5 shows gene rankings in the basis of our mixed model 3, the NormFinder Model-Based method [5] and the geNorm Pair-Wise comparison method [4]. For the geNorm approach, the stability values M calculated by this software for each gene was used to establish the ranking. Spearman rank correlation between classifications from mixed model 3 and from the other two procedures was 0.66 for NormFinder and 0.62 for geNorm. Rank correlation between NormFinder and geNorm classifications was 0.79. Although the three rankings were different, some coincidences were found in the first five positions. Thus, *RPS18, SDHA *and *B2M *were shared among all methods, while *ACTB *and *YWHAB *only between two of them. Similarly, *ACACA, ATP5G2 *and *RPL19 *were among the least stable genes under the three approaches. On the other hand, the position of some genes (i.e. *YWHAB *and *RPL13A*) was considerably different depending on the approach.

**Table 5:** Ranking of genes based on ML-mixed model 3, NormFinder and geNorm approaches.

Ranking position	gene	ML-Mixed model 3	gene	NormFinder	gene	geNorm
best pair	RPS26/SDHA	0.0005	RPS26/YWHAB	0.008	ACTB/RPL13A	0.294

1	RPS18	0.0007	RPS18	0.013	YWHAB	0.782

2	RPS26	0.0009	B2M	0.022	RPS18	0.790

3	SDHA	0.0011	YWHAZ	0.024	ACTB	0.796

4	B2M	0.0015	SDHA	0.026	SDHA	0.797

5	ACTB	0.0017	YWHAB	0.029	B2M	0.815

6	MDH1	0.0019	RPS26	0.030	YWHAZ	0.833

7	YWHAZ	0.0020	ACTB	0.035	RPL13A	0.839

8	LOC780524	0.0027	CYP1A1	0.037	RPS26	0.847

9	GAPDH	0.0030	GAPDH	0.039	GAPDH	0.882

10	YWHAB	0.0033	RPL13A	0.043	RPLP0	0.899

11	RPLP0	0.0038	MDH1	0.048	LOC780524	1.085

12	RPL19	0.0039	ACACA	0.057	MDH1	1.119

13	ATP5G2	0.0057	RPLP0	0.058	RPL19	1.140

14	CYP1A1	0.0063	LOC780524	0.061	CYP1A1	1.170

15	ACACA	0.0069	RPL19	0.072	ATP5G2	1.308

16	RPL13A	0.0078	ATP5G2	0.077	ACACA	1.568

**Figure 2:**
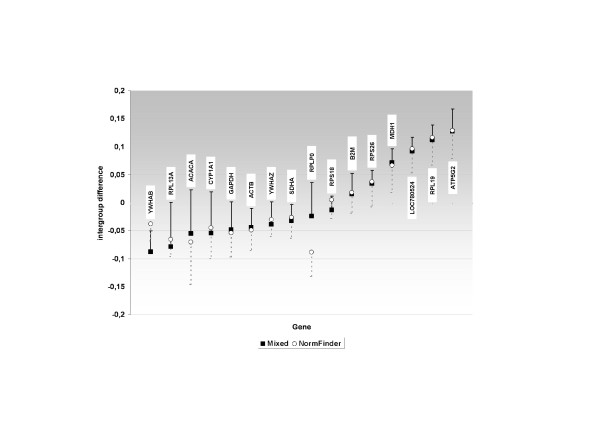
**Comparison between NormFinder and ML-mixed model**. Comparison between NormFinder and ML-mixed model 3 estimates of inter- and intra-group variability. Inter-group variation is represented in the Y axes. Intra-group variation (bias from our ML-mixed model procedure and intra-group standard deviations from the NormFinder one) is presented as error bars on estimates of the inter-group differences for each gene. Downward dotted bars correspond to NormFinder estimates and upward solid bars to ML-mixed model 3.

NormFinder and mixed model 3 are further compared in Figure 2, where inter- and intra-group variabilities are presented for both procedures. With the exception of *YWHAB *and *RPL13A*, inter-group differences (control-heat stress) estimated following both procedures were similar for most of the genes. Those genes which were over-expressed in heat stress (i.e. *ACACA, CYP1A1, GAPDH*) conditions, showed negative inter-group differences. Conversely, those genes that were down-regulated in heat stress (i.e. *LOC780524, ATP5G2 *and *RPL19*) showed positive inter-group differences. It is important to highlight that the simultaneous use of genes with positive and negative values would yield lower MSE and NormFinder stability values than sets involving genes with the same sign, due to compensating effects.

### Optimal set of RGs

Best pairs of stable genes for all approaches are shown in the first line of Table 5. In the geNorm approach, stability value of the best pair of genes corresponded to that obtained after several steps of stepwise exclusion of the least stable gene until only two genes from the whole set of sixteen remain. These pairs were different in every approach, *RPS26*/*SDHA *for mixed model 3, *RPS26*/*YWHAB *for NormFinder and *ACTB*/*RPL13A *for geNorm. Under mixed model 3, average MSE for all possible combinations of genes from 2 to 16 (65.519 combinations) were explored to determine the best set of stable genes to be used as normalizers. Table 6 shows the average MSE of the best combinations of sets from 2 to 7 genes and also for the set including all genes. The *RPS18*/*RPS26*/*SDHA *set had the lowest average MSE (0.00043), revealing the best combination of normalizers. Similarly, *RPS26/SDHA *and *RPS18/SDHA *pairs had average MSE values of 0.00047. Additional file 1 shows the optimal number of control genes for normalization estimated by the geNorm. geNorm authors [4] recommend a cut-off value of 0.15, thus, the group of two genes (0.100) was the best combination (see 2 vs. 3 genes).

**Table 6:** Average MSE of the best genes combination for sets from 2 to 7 genes tested under ML-mixed model 3

Gene set							Average MSE
2	RPS26	SDHA					0.00047

120*	RPS18	SDHA					0.00047

	MDH1	SDHA					0.00057

	B2M	ACTB					0.00077

	MDH1	ACTB					0.00096

	SDHA	ACTB					0.00099

	B2M	SDHA					0.00116

	B2M	MDH1					0.00122

3	RPS18	RPS26	SDHA				0.00043

560	RPS18	LOC780524	YWHAB				0.00050

	RPS18	MDH1	YWHAB				0.00050

	RPL19	ACTB	SDHA				0.00057

	MDH1	SDHA	ACTB				0.00063

	MDH1	B2M	ACTB				0.00068

	MDH1	SDHA	B2M				0.00087

	SDHA	ACTB	B2M				0.00088

4	RPS18	RPS26	MDH1	YWHAB			0.00051

1,820	RPS18	RPL19	ACTB	SDHA			0.00051

	RPS18	RPS26	LOC780524	YWHAB			0.00052

5	RPS18	RPS26	LOC780524	SDHA	YWHAB		0.00048

4,368	RPS18	RPS26	MDH1	SDHA	YWHAB		0.00049

	RPS18	RPS26	B2M	ACTB	SDHA		0.00051

6	RPS18	RPS26	MDH1	LOC780524	SDHA	YWHAB	0.00049

8,008	RPS18	RPS26	RPL19	ACTB	SDHA	YWHAB	0.00052

	RPS18	MDH1	RPL19	ACTB	SDHA	YWHAB	0.00053

7	RPS18	RPS26	MDH1	RPL19	ACTB	SDHA	0.00048

11,440	RPS18	RPS26	LOC780524	RPL19	ACTB	SDHA	0.00049

	RPS18	MDH1	LOC780524	RPL19	ACTB	SDHA	0.00054

All genes							0.00156

*Under each set number (from 2 to 7) the number of possible combinations appears.

## Discussion

### ML-Mixed model approach

The stability ranking generated by mixed model 3 (Table 5) placed *RPS18, RPS26, SDHA, B2M *and *ACTB *in the top five positions. However, as previously mentioned, the selection of RGs has to be based not only on the estimated stability of gene expression, but also on biological and functional criteria. For instance, despite the fact that *RPS18 *showed a high stability, some works indicate that it is not a good control gene [15,16] because its transcription is carried out by RNA polymerase I, and for accurate quantification by qPCR the use of RGs following the same type of transcription pathways is important. *RPS26 *encodes the 40S ribosomal protein S26. S26 mRNA half-life is more than 20 hours and was found to be expressed at high and comparable levels in various adult human tissues [17]. However, *RPS26 *has multiple processed pseudogenes dispersed through the genome [18,19]. Primers are usually designed to overlap two or more exons in order to distinguish PCR products derived from genomic DNA and RNA. If some of these pseudogenes are expressed either constitutively or under particular circumstances, several sequences from different genes could be co-amplified leading to wrong results.

*SDHA *encodes the succinate dehydrogenase complex subunit A flavoprotein which converts succinate to fumarate as part of the Citric Acid Cycle. Under mixed model 3 this gene showed similar and small variances in control (0.00006) and heat stressed (0.00087) samples, appearing in the 3^rd ^position of the stability ranking. This result is in agreement with other studies which have pointed out *SDHA *as a good RG to be used in different circumstances, including heat stress in several species, [20]. Beta-2 microglobulin is a component of MHC class I molecules gene and is encoded by *B2M*, which has been extensively used as endogenous control in many publications. However, its expression varies considerably under different experimental conditions and therefore its use for normalization is limited. Under mixed model 3, *B2M *showed higher variance in heat stress conditions (0.00142) than under mild temperatures (0.00008). However since its bias was among the lowest (0.0075), it can be considered as a good RG to study heat stress in ovine blood samples.

*ACTB *is another commonly used RG in qPCR assays. Its expression has been shown to vary in a tissue-specific and time-dependent manner [12]. However, our results are in line with other studies reporting stable expression of *ACTB *under temperature stress in *Orchesella cincta *[11]. Although under our ML-mixed model approach *ACTB *is placed among the top five stable genes, this was not the case when comparing sets of two or three genes.

Conversely, *MDH1 *took the 6^th ^place in the mixed model 3 stability ranking, but appeared among the genes constituting the most stable sets. *MDH1 *encodes the cytosolic malate dehydrogenase which catalyzes the reversible oxidation of malate to oxaloacetate, utilizing the NAD/NADH cofactor system in the citric acid cycle. Cellular localization studies indicate that *MDH1 *mRNA expression has a strong tissue-specific distribution, being expressed primarily in cardiac and skeletal muscle and in the brain, at intermediate levels in the spleen, kidney, intestine, liver and testes and at low levels in lung and bone marrow [21]. This study demonstrated that *MDH1 *expression was stable under different temperature conditions in ovine whole blood samples, and points toward this gene as a suitable RG to be used in expression studies addressing the heat stress response in sheep.

### Comparison of methods

The top five positions of the stability rankings obtained with the mixed model 3, NormFinder and geNorm approaches (Table 5) contained the *RPS18, SDHA *and *B2M *candidates. This shows the accordance of the three methods to identify highly stable genes even when different calculation methods are used. However, for the remaining genes there were many larger differences among the three rankings.

Model based procedures such as the ones proposed by Andersen et al. [5-7] and Abruzzo et al. [6] are appealing. They provide a solid statistical framework which allows accounting for different sources of variation (e.g. differences in expression levels across genes, amount of mRNA in the samples, treatments, tissues and cells types, developmental stages, sampling, etc.) in the estimation of the genes stability. The goal is to achieve a prediction of gene stability invariant to a variety of effects, making the set of selected RGs more generally applicable. In our case, a model including sample and its interactions with treatment and gene was deemed more plausible for the data analyzed (using likelihood based criteria) and for data not used in the analysis (using cross validation statistics). Accounting for those factors when estimating the parameters of interest (gene by treatment effect and gene-treatment variances in our case) removes noise and provides estimates free of the effects of the specific animals sampled for this experiment. The model underlying the NormFinder application [5] does not consider the interaction between sample and treatment and showed a worse predictive ability, illustrating the interest of adjusting for noise to obtain estimates of gene stability applicable to future samples.

Within the model based methods, several alternatives have been proposed to obtain the stability measure. In NormFinder [5], inter-group variation is computed as the least square estimate of the group by gene interaction and intra-group variability is obtained following the method of moments approach. Those two components are closely linked to the bias and variance components of the MSE calculation of Szabo et al. [6], followed in our study. Figure 2 shows inter-group variation (from NormFinder) and bias (from our ML-mixed model procedure) for all genes, together with the intra-group standard deviations from NormFinder and from our procedure represented by error bars. Except for *RPLP0 *and *YWHAB*, the inter-group variability estimate obtained by the two procedures was similar for all genes (Pearson correlation coefficient = 0.95). However, the intra-group variability was more dissimilar (Pearson correlation coefficient = 0.38). This may be explained by the different estimation methods followed in both procedures (maximum likelihood in our approach and method of moments in NormFinder [5]). Maximum likelihood estimates have well known asymptotically optimal properties in terms of bias and variance while unbiasedness is the only optimal property of methods of moments. Andersen et al. [5] argued that using REML to obtain estimates of intra-group variances yielded similar results in their data set to the method of moments proposed, but, this result cannot be generalized. Another difference between the stability value obtained by NormFinder and the MSE stability value [6] used in our study is how the inter- and intra-group variation are combined. In NormFinder, those two components are combined by adding estimates of the mean and standard deviation of the posterior distribution (following Bayesian terminology) of the deviation of the observed group mean for each gene from the overall expected value for the log-transformed measure of the gene expression. The mean and variance of the distribution of that deviation depends on inter- and intra-group variation, respectively. This results in a stability value where inter- and intra-group variability do not have the same weight. This is in contrast with the MSE situation, where these two components are equally weighted. Nevertheless, when intra-group variability is close to zero, the weights on both components are equal. In our data set, the estimated intra-group variances for all genes are small (as expected for RGs). We obtained a stability measure by adding the inter-group variation and the average of the square root of the intra-group variations provided in the extended output of NormFinder which was similar to the overall stability value provided by that software (Pearson and Spearman correlations = 0.96).

The use of a more solid statistical framework such as our ML-mixed approach allows for statistical testing of the differences in gene expression across different conditions. This is an alternative way and a double check through statistical validation procedures for the selection of stable genes, as recently recommended Setiawan and Lokman [22]. In our case, a t-test on the estimated gene by treatment effects provided by the SAS mixed models procedure (PROC MIXED) indicated that differences in expression between the two treatments were only significant (p < 5%) for *RPL19 *and *ATP5G2 *which were positioned by the three procedures among the five most instable genes and considered together with *ACACA*, among the worst. This shows consistency between stability values and statistical validation approaches.

The difference between our procedure and the one followed by Szabo et al. [6] is that the gene by group interaction is treated as a random effect instead of as a fixed factor. Random effects represent random deviations from the expected value of each data due to that effect. In our case, the random treatment by gene interaction can therefore be used as a direct measure of the so called bias in [6].

Compared to model based procedures, the pair-wise comparison approach used in geNorm [4] is easier to apply and does not require normality assumptions (while maximum likelihood model based methods do). Furthermore, it has shown more robust behavior than NormFinder with small sample sizes [23]. Nevertheless, geNorm has the tendency to top rank genes with correlated expression profiles rather than with minimal variation [5] and does not accommodate the existence of different groups of measures in the calculation of the stability value. When different groups of samples exist, independent analyses for each set of data have to be carried out, often yielding different optimal sets of RGs in each group. We have run geNorm for each set of data (control and heat stress) separately (data not shown). Ranking of RGs differed between treatments, but, two genes, *SDHA *and *B2M *were shared among the top five of both groups. A better agreement was found for the least stable genes (*ACACA, ATP5G2, RPL19 *and *CYP1A1)*, which were located in both treatments in the last five positions. Alternatively, the procedures proposed by Andersen et al. [5] and our ML-mixed model approach can also provide within group stability values. Ranking differences were also observed for these procedures between treatments. Here the differences were also bigger among the most stable genes than among those genes estimated as the less stable ones. Results from the different methods were more similar in the control (thermo neutral) than in the heat stress environment. Notice that within groups, differences in stability among genes are only associated to changes in intra-group variances. Genes such as *B2M *and *SDHA*, which were classified by our ML-mixed model approach within the top five stable genes, both globally and in the control group, showed larger variability under heat stress, and were classified as the less stable ones under heat conditions. Since in many situations the goal is to use a given set of RGs in the normalization of the expression of target genes across different conditions, an essential requirement for the RGs is stability of their transcriptional levels across those conditions. The within group stability will only be relevant when target genes are compared under the same experimental conditions.

Finally, one constraint of the geNorm and NormFinder softwares come from the fact that they are implemented for the Microsoft Excel application which involves some restriction regarding the number of samples, treatments and genes that can be analyzed simultaneously. Furthermore, the geNorm application, does not allow empty cells, therefore, those samples in which the amplification of one or more genes fails must be eliminated from the analysis.

### Best set of RGs

There is no consensus about the amount of RGs that should be used in expression studies [4,24,25], although in all cases the common objective is to find the best alternative regarding accuracy and technical constraints. We have analyzed in this work all possible sets of genes considering, both stability and biological criteria. As explained above, *RPS18 *and *RPS26 *have been discarded from the analysis for functional reasons. With the ML-mixed model 3 the best set of two genes was *SDHA/MDH1 *(MSE = 0.0006) and the best one of three genes was *RPL19/ACTB/SDHA *(MSE = 0.0006), after discarding these genes. These values were very close to those of the best pair *RPS26/SDHA *(MSE = 0.0005) obtained under our ML-mixed model 3 approach when all candidates were included. However, pairs constituted by *SDHA*/*B2M *and *SDHA*/*ACTB *had larger MSE values (0.0012 and 0.0010, respectively). For the geNorm approach the best pair of genes was the same, *ACTB/RPL13A*, as the one obtained when including all candidates. However, NormFinder selected a different pair of genes *B2M/SDHA*. One again, ML-mixed model 3 and NormFinder approaches identify the same gene (*SDHA) *for the best set of two genes.

### Normalization factors and normalized targets

Normalization factors were calculated by means of the Delta Ct method [4] for the best pair of reference genes selected with each statistical approach, *ACTB/RPL13A*, *B2M/SDHA *and *SDHA/MDH1 *for geNorm, NormFinder and ML-mixed model, respectively (Additional file 2). Two of the less stable genes *CYP1A1 *and *ACACA *were used as targets to study the impact of using different nornalizers. Pearson and Spearman correlations estimates among normalized values of targets ranged from 0.80 to 0.92 for geNorm-NormFinder, from 0.67 to 0.89 for geNorm-ML- mixed model and from 0.92 to 0.98 for NormFinder-Ml mixed model. Model based methods, NormFinder and ML-mixed model showed the highest correlations indicating statistical similarities underlying the estimation of genes stability under both methods. Although correlations among results obtained under all approaches were high, small differences among the normalized expression rates of targets might be critical when the aim is to detect differential expression among samples from different treatments, tissues, cell types or genotypes.

## Conclusions

A ML-mixed model approach has been presented here as a suitable method to select stable genes to be used as RGs in gene expression studies. Optimal statistical properties of ML estimation together with the flexibility of the mixed model allows estimation of gene expression stability under many different situations without constraints in the amount of data, number of genes and number of treatments or tissues tested. A model selection step can also be performed to choose the optimal model to estimate stability values. The use of goodness of fit and predictive ability criteria is recommended because they measure different quality criteria and can provide unequal model rankings.

Although in the present study we have tested several mixed models according to our experimental needs, many other possibilities can be considered to take into account for new biological situations. For instance, in situations such as drug competition in which interaction between treatments must be fitted, or experiments of sequential gene expression along a period of time where the existence of correlation between successive samples needs to be considered by fitting some structure among residuals.

Comparison with two other procedures currently used showed differences in genes ranking according to their stability values, which were mainly explained by the difference in estimates for the within treatment variability. Also differences in normalized expression values of targets were found among the three methods tested. Nevertheless, some genes were selected by the three approaches. *RPS18, SDHA *and *B2M *were ranked in the first five positions by the three methods. On the last positions, *ACACA, ATP5G2 *and *RPL19 *were the least stable genes under the three approaches.

In this work, the pair of genes *SDHA/MDH1 *is recommended to normalize target genes expression in peripheral whole blood in studies of the heat-stress response in sheep.

## Methods

### Selection of RGs

Sixteen candidate RGs were tested. Twelve of them (*RPS18 (*Ribosomal protein S18), *YWHAZ (*Tyrosine 3-monooxygenase/tryptophan 5-monooxygenase activation protein, zeta polypeptide), *ACACA (*Acetyl-CoA carboxylase), *RPS26 (*Ribosomal protein S26), *B2M (*Beta-2 microglobulin), *MDH1 (*Malate dehydrogenase), *LOC780524 (*Ribosomal protein S2, RPS2), *RPL19 (*Ribosomal protein L19), *ATP5G2 (*ATP synthase, H+ transporting, mitochondrial Fo complex, subunit C2, subunit 9), *GAPDH (*Glyceraldehyde 3-phosphate dehydrogenase), *CYP1A1 (*Cytochrome P4501A1) and *ACTB (*Actin beta)) were included in the Sheep geNorm kit (Primerdesign Ltd, UK). The remaining four genes (*RPL13A (*Ribosomal protein L13a), *RPLP0 (*Ribosomal protein large P0), *SDHA (*Succinate dehydrogenase complex, subunit A, flavoprotein) and *YWHAB (*Tyrosine 3-monooxygenase/tryptophan 5-monooxygenase activation protein, beta polypeptide)) were choosen because they had been tested as RGs in a gene expression study of the ovine *PRNP *and *SPRN *[13] genes. Genes, GeneBank accession numbers and gene ontology are listed in Table 7.

**Table 7:** Tested candidate reference genes

Gene symbol	Gene full name	Gene ID	GO
*RPS18*	Ribosomal protein S18	100036761	Translation

*YWHAZ*	Tyrosine 3-monooxygenase/tryptophan 5-monooxygenase activation protein, zeta polypeptide	780452	mRNA metabolic process

*ACACA*	Acetyl-CoA carboxilase	443186	Lipid process

*RPS26*	Ribosomal protein S26	443468	Translation

*B2M*	Beta-2 microglobulin	443295	Immune response

*MDH1*	Malate dehydrogenase	443091	Glycolysis

*LOC780524*	Ribosomal protein S2 (RPS2)	780524	Translation

*RPL19*	Ribosomal protein L19	100270789	Translation

*ATP5G2*	ATP synthase, H+ transporting, mitochondrial Fo complex, subunit C2 (subunit 9)	443542	Ion transport

*GAPDH*	Glyceraldehyde 3-phosphate dehydrogenase	443005	Glycolysis

*CYP1A1*	Cytochrome P4501A1	100170113	Oxidation-reduction process

*ACTB*	Actin beta	443052	Cellular component movement

*RPL13A*	Ribosomal protein L13a	100036760	Translation

*RPLP0*	Ribosomal protein large P0	100036764	Translation

*SDHA*	Succinate dehydrogenase complex, subunit A, flavoprotein	100036762	Oxidation-reduction process

*YWHAB*	Tyrosine 3-monooxygenase/tryptophan 5-monooxygenase activation protein, beta polypeptide	100036763	Protein targeting

### Animal samples, total RNA isolation and cDNA synthesis

Peripheral whole blood samples from 15 rams of the Manchega Spanish sheep breed were collected under two different climatic conditions in a dry region of central Spain (Ciudad Real). The control samples were collected from 15 animals with an environmental temperature of 28.6°C and a relative humidity of 52%. The second set of samples, here considered as the heat stress conditions, was collected from the same animals at an environmental temperature and a relative humidity of 34.4°C and 35%, respectively (data from Ciudad Real Meteorological Station, coordinates 629 m-38 59N-03 55W).

Total RNA was isolated from 10 ml of whole blood using the LeukoLock kit (Ambion, Inc., TX, USA) following manufacturers instructions. The absence of DNA contamination was verified by minus RT control PCR. The quality of the RNA was assessed based on the demonstration of distinct intact 28S and 18S ribosomal RNA bands. RNA concentration was determined using a NanoDrop ND-1000 UV/Vis spectrophotometer (Nanodrop Technologies, Inc., DE, USA).

cDNA was synthesized using the ImProm-II™ Reverse Transcription System (Promega Corp., WI, USA). In a first step the primer/RNA mix [RNA (500 ng), oligo (dt)_15 _primer (0.5 μg/reaction) and random primers (0.5 μg/reaction) in a final volume of 8 μl] was heated 5 min at 70°C and quickly chilled on ice for 5 min. In a second step, the reverse transcription mix [ImProm-II™ 5× reaction buffer (1.6×), MgCl_2 _(2.8 mM), dNTP mix (0.6 mM each dNTP), 10U of Recombinant RNAsin Ribonuclease Inhibitor and 20U of ImProm-II™ Reverse Transcription in a final volume of 12 μl] was mixed with the primer/RNA mix in a final volume of 20 μl and subjected to the following thermal profile: 5 min annealing at 25°C, 60 min first-strand synthesis reaction at 42°C and 15 min inactivation of reverse transcriptase at 70°C. The final product was stored at -20°C.

### qPCR

qPCR amplification reactions were performed in a final volume of 20 μl containing 50 ng of cDNA, 10 μl of Precision ™ 2X qPCR Mastermix with SYBR GREEN (Biomolecular Technologies, Inc., USA) and 300 nM of each primer. Reactions were run in triplicate on an ABI PRISM 7500 Fast Sequence Detector (Applied Biosystems, CA, USA) following the manufacturer's cycling parameters. Dissociation curves were performed for each gene to check primer specificity and to confirm the presence of a unique PCR product. The corresponding mRNA levels were measured and analyzed by the 7500 System Software (Applied Biosystems, CA, USA). The Cq (threshold cycle) is defined as the number of cycles needed for the fluorescence to reach a specific threshold level of detection and is inversely correlated with the amount of RNA template present in the reaction.

To estimate PCR efficiencies, standard curves based on 5 serial dilutions (0.08, 0.4, 2, 10 and 50 ng/μl) of a cDNA stock (a cDNA mixture of all samples collected) were also performed. Efficiencies (E) were calculated from the slope of curves using the formula E = 10^(-1/slope) ^[26,27]. For all candidate genes plates consisted of 87 wells, derived from 15 RNA samples (one RNA isolation was lost) for each thermal treatment, each with three technical replicates.

### Statistical data analysis. Maximum Likelihood Mixed Model approach

The use of efficiency corrected mathematical models are strongly recommended and leads to more reliable estimates of the 'real expression ratio' compared to non efficiency corrected ones [28]. In order to take the differences in the amplification efficiency (E) of the qPCR reaction of each gene into account, the log_E _of the raw Cq data was used as the dependent variable. In this regard, it is important to consider that a commonly used approach is to asume 100% efficiency (E = 2) for all genes. Replicates were included in the model as repeated measures of the same animal which allows for a better correction of technical differences in well's loading.

Different Mixed models were fitted using the MIXED procedure of the SAS/STAT^® ^statistical package. Models included treatment (t) and gene (g) as systematic fixed effects; sample (a), treatment × gene (tg), treatment × sample (ta) and gene × sample (ga) as alternative random variables, and homoskedastic (equal residual variance for all observations) and heteroskedastic (different residual variances for groups of observations pertaining to each class of either the gene or the treatment × gene effects) residuals. The model proposed by Andersen and coworkers [5] which includes tg and ta as fixed effects and a heteroskedastic residual variance for the tg effect was also tested (model 8). Table 3 shows the equations of the mixed models tested, their variance structure and the number of (co)variance parameters.

The estimation method was Maximum Likelihood (ML). Several statistics were used as Goodness of Fit indicators: -2 Log Likelihood (-2logL, smaller is better), Akaike's Information Criterion (AIC = -2l+2d, smaller is better), and Schwarz's Bayesian Criterion (BIC = -2l+dlogn, smaller is better), where l is the maximum value of the log likelihood, d is the dimension of the model and n is the number of effective observations. Model Predictive Ability was evaluated by crossvalidation using a training set (for estimation of parameters) composed of 917 records (2/3 of the whole data available) and a testing set (for prediction of new data) with 460 records (1/3 of the original set of observations). Average square differences (PD) between observed and predicted values were used as models predictive ability criterion. PD% reflects the percentage of predictive ability loss (smaller is better).

The measure of the gene expression stability was obtained by calculating the Mean Square Error (MSE), as in [6] under the best model selected in the previous step. MSE was defined as:

$$\text{MSE}={\left(\text{bias}\right)}^{\text{2}}+\text{variance}$$

In this equation, bias was the estimate of the random treatment × gene interaction and variance was the ML estimate of the variance of the residual term corresponding to the selected model.

To rank genes on the basis of their stability, the minimax MSE criterion of Szabo et al. [6] was performed. In brief, the gene with the smallest value among the largest MSE of the two treatments was selected as the most stable one. To find a set of genes for normalization across treatments, all possible combinations of genes from 2 to 16 were explored. For every combination, MSE was calculated by averaging bias and variances of the genes contained in each set.

### Other approaches currently used

In order to contrast the results obtained under our statistical approach to test candidate RGs stability, we run the geNorm [4] and the Normfinder [5] softwares. As both programs rely on the input of relative values, the Cq of the 3 replicates per sample were averaged in a single value.

For the geNorm approach, sample Cq values were transformed to relative quantities (Q) with the equation:

$$\text{Q}={\text{E}}^{\left(\text{minCq}-\text{sampleCq}\right)}$$

where E = amplification efficiency; minCq = lowest Cq value (Cq value of the sample with the highest expression) and sampleCq = Cq value of each sample. geNorm is based on the principle that the expression ratio of two ideal RGs is identical in all samples, and defines the *M *value as the average pair-wise comparison of a gene with all the other tested. Genes with low *M *values have less variation and more stable expression. Then, the genes are ranked according to their expression stability *M *value. geNorm does not accommodate the existence of groups (treatments in our case) and therefore samples obtained under heat stress or thermoneutral conditions were treated alike.

For NormFinder, the dependent variable was the log_E _of the Cq values as in our ML-mixed model approach. Genes are ranked according to a stability value that combines intra- and inter- group variability obtained from a fixed effects model.

Both softwares were used to determine a ranking of gene expression stability, in order to compare them with the results obtained under our mixed model approach.

Normalization factors were calculated using the Delta Ct method [4] for the best pair of reference genes selected by each approach tested. Expression values of target genes were normalized to test differences from the reference genes used.

## Authors' contributions

MS conceived of the study, performed the statistical analyses and draft the manuscript. NMS participated in qPCR data analyses and in drafting the manuscript. CG carried out the qPCR assays. AMC has participated in the qPCR assays design, protocols development and in drafting the manuscript. MVP has participated in the qPCR assays design, protocols development and software management. JHC contributed in the experimental design and in results interpretation. JS has processed biological samples and contributed to qPCR assays. JC has contributed to qPCR assays, standard curves design and discussion. MJ participated in statistical analyses design, results interpretation and in drafting the manuscript. All authors read and approved the final manuscript.

## Supplementary Material

Additional file 1 **GeNorm optimum number of reference genes**. Evaluation of the optimum number of reference genes according to the geNorm software. The magnitude of the change in the normalization factor after the inclusion of an additional gene reflects the improvement obtained. Click here for file

Additional file 2 **Normalization factors**. Normalization factors calculated with the Delta Ct method for each of the best pair of reference genes selected by geNorm, NormFinder and ML-mixed model 3. Click here for file
