# Supplementary figures and images for: Use of Maximum Likelihood-Mixed Models to select stable reference genes: a case of heat stress response in sheep
Source: BMC Mol Biol. 2011 Aug 17;12:36. doi: 10.1186/1471-2199-12-36 (PMC3175163; doi:10.1186/1471-2199-12-36)

### Determination of the optimal number of control genes for normalization

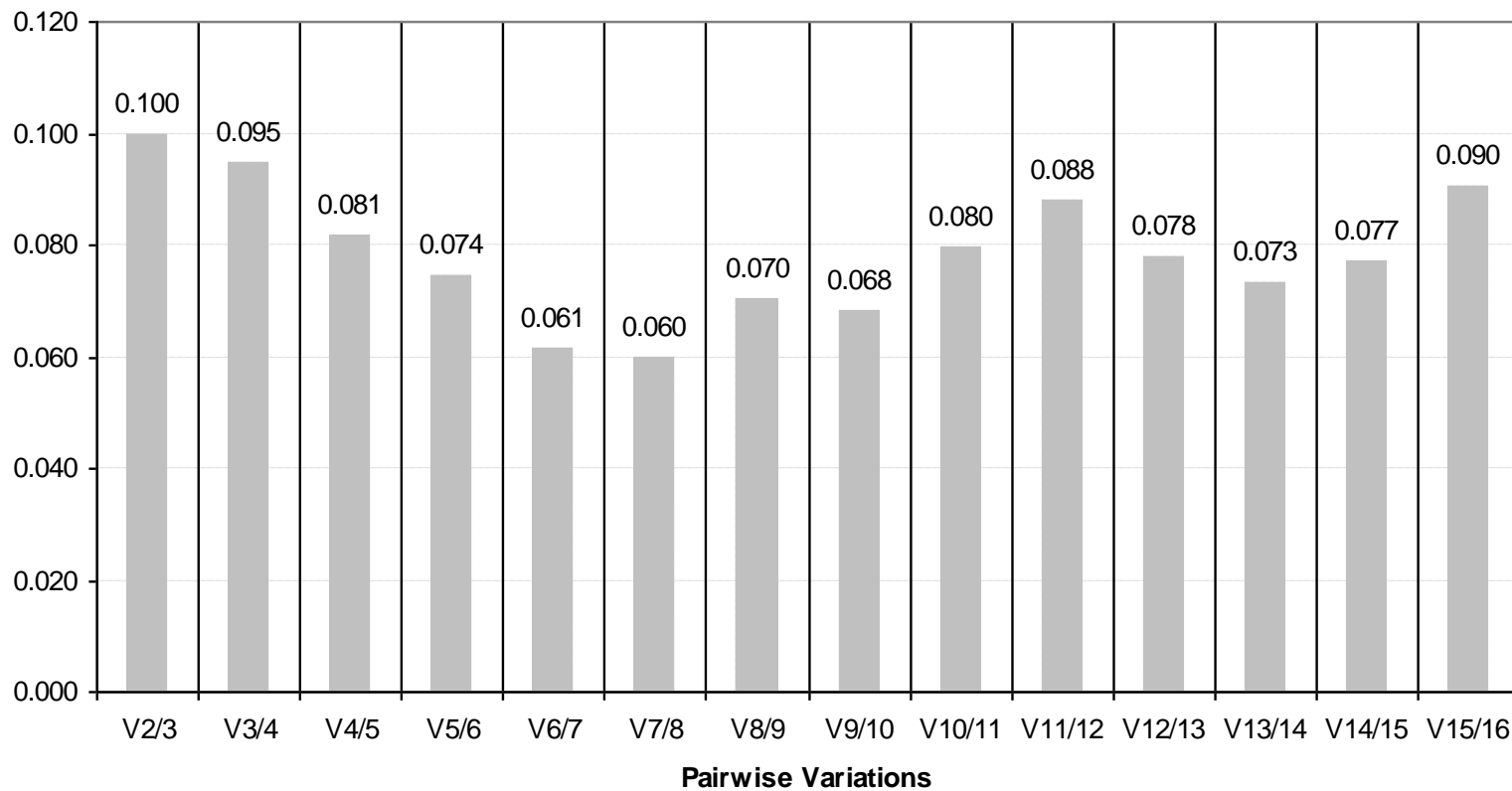

Supplement: Additional file 1 — GeNorm optimum number of reference genes. Evaluation of the optimum number of reference genes according to the geNorm software. The magnitude of the change in the normalization factor after the inclusion of an additional gene reflects the improvement obtained. [file 1471-2199-12-36-S1.PDF]
